# ‘Drawing out the Whole Picture’: Positive and Gestalt Effects of Taking Sign-Based Notes on Listening Performance in Chinese ESL Classrooms

**DOI:** 10.3390/bs13050395

**Published:** 2023-05-09

**Authors:** Minmin Yang, Gretchen McAllister

**Affiliations:** 1 College of Foreign Languages, Huaqiao University, Quanzhou 362021, China; 2 College of Education, Northern Arizona University, Flagstaff, AZ 86011, USA

**Keywords:** note-taking, sign-based notes, gestalt, L2 listening

## Abstract

The effort to design a most ideal strategy for L2 learners to take notes in L2 (EFL/EMI/EAP) classrooms has received growing attention. However, note-taking has been repeatedly tested and reported diverging impacts on students’ learning. This study investigates the effects of sign-based note-taking (SBN) with the traditional way of using pens and paper, and it features the cognitive processes of understanding and creating notes. SBN guides students to comprehend and draw a gestalt of notes using signs (i.e., icons, indices, and symbols). In a 16-week mixed study, three types of interventions—a traditional treatment, TOEFL’s ‘good-note guidance’ (GNG), and SBN—were administered to three separate student groups, namely a control group (CG) and two experiment groups (EG1 and EG2). Pre-, post-, delayed tests, questionnaires, and post-intervention interviews were conducted and analyzed for the needs and the effects of interventions on listening performances. Findings are as follows: only EG2 achieved significantly higher performance regardless of instructor’s influence, proving gestalt-based SBN an effective cognitive practice; GNG improved performance over time; students favored SBN, wanting longer-duration guidance. These results confirm that gestalt strengthens memory for L2 listening and yields pedagogical implications for L2 Listening classrooms.

## 1. Introduction

Listening comprehension is important as adults spend half of their communication time listening. Second language learning, L2, listening has been under-researched, particularly when compared to other skills of language learning. Historically, it has proven to be challenging to research, as instructors find it difficult to teach and listeners find it challenging to acquire [1,2]. Challenges in L2 Listening have been noted, and attempts to address them have been made using a variety of strategies [2]. From as far back as 100 years ago, Crawford (1925) [3] conducted experiments to test and confirm the effects of note-taking on listening comprehension, to the present research still examining note-taking’s role in improving academic acquisition [4,5,6,7,8], note-taking has generally been recognized as a “component crucial to learning in academic contexts” [9] and even “THE distinguished characteristic of higher education” [10].

Among all L2 strategies, note-taking is especially controversial, difficult, inclusive, and extensive [2] (p. 579). The note-taking research covers several areas that include: (1) lecture characteristics, (2) note-taking methods, (3) individual differences, and (4) testing procedures [11], and the practice of it concerns at least “what/how” to teach from the teachers’ perspective and “what/how” to learn and do from the students’ perspective. It is literally “controversial” in that diverse research yields diverging conclusions. While some researchers find that note-taking does not significantly affect listening performance [12] (p. 29), [13] (p. 1), many other researchers offer proof of a positive, albeit modest, correlation between such practice and performance [14,15]. This study uses a mixed-methods approach to investigate the efficacy of three notetaking methods, with the inclusion of a new approach, SBN, designed by one of the authors, within an L2 classroom. This includes interviews, as well as a 3 × 3 intervention to test the notetaking models’ effects.

The effectiveness of listening comprehension is crucial for not only classroom settings, but also for interpersonal and cross-cultural communication. Though this research is conducted in the L2 context, we believe that this study’s findings will extend from English as Foreign Language to studies of other languages, to higher education learning contexts, as well as to the discipline of communication. By presenting and testing an innovative note-taking SBN approach, the cognitive perspective this study takes may also contribute to the growing body of behavioral science experiments offering empirical proof of pedagogical innovations.

### 1.1. Note-Taking Research in L2 Listening: From Format to Content

With regard to teaching students note-taking, copious research has focused on the success of ‘guided notes’, which primarily concerns how notes should be formatted. There is a certain consensus that an outline format is optimal and that the instructor providing students with practice using the lecture format is beneficial [15]. The formats usually include a structured outline or some other forms of organizers, attaching basic background information with standard cues and space for students to write out key points. Tsai and Wu (2010) adopted the most classic choice of a Cornell format in their intervention, finding that this format is helpful in producing a higher score of listening tests, regardless of the language the Chinese speaking L2 learners use (L1 or L2) [16]. A recent study using the Cornell format also verified that it is beneficial for the Korean EFL learners in comprehension tests [17]. The Cornell system is also often introduced in commercial textbooks [9] (p. 247). It appears, then, that many format-targeted studies have been re-testing what had been proven positive nearly 100 years ago: that an intervention or instruction of format is helpful for students.

Nevertheless, formats do not always yield such conclusions and students may not always favor a particular format. In Bui et. al.’s study (2013), when participants were allowed to revisit their notes, those who had tried to transcribe the lecture outperformed, as measured by delayed tests, those who had taken organized notes [18]. That seems to suggest that when compared with other methods of note-taking, the function of a specific format is likely to be challenged.

Bui and McDaniel (2015) contrasted the effects on test performance and free recall after listening to a lecture of different learning aids: a skeletal outline, an illustrative diagram, or no aid at all [19]. Experiment results show that a group of low-ability structure builders who were given the skeleton outline produce enhanced free recall. However, they did not exhibit better short-answer performance than the group that received no aid at all. The results, though, were more optimistic for the high-ability structure builders. Contrastingly, a striking finding is that if given an illustrative diagram, the results on both tests turn out positive regardless of the level of the learner’s structure-building abilities.

Despite the diverging findings, previous format-focused studies have addressed the question of whether a particular technique will enhance listening skills in L2 users. But there are additional contextual challenges that must be addressed as well. A format may help with particular exercises, but it is not realistic in most exam or authentic successive listening situations. This is because guides and formatted note-paper are not typically utilized in exams or in real-life listening environments. Therefore, while a diagram in Bui and McDaniel’s design (2015) might function very well, despite a learner’s individual differences, there is no practitioner available to suggest/offer such a diagram to L2 listeners [19]. Furthermore, it has been claimed that the format has been less convincing for the recording of individual items [9] (p. 251). Therefore, it seems reasonable to switch the focus of teaching note-taking from format to content.

Concerning the content of notes, many teachers simply instruct students just to “take notes”, without explicit instruction. Such lack of explicit instruction surely will not be helpful, especially when compared with explicit instruction [20]. Some other teachers will instruct students to take down key words. Most EFL listeners, however, frequently mistake ‘key words’ for ‘items of vocabulary that they happened to understand or hear’ [2] (p. 757). Students’ insensitivity to key words leads instructors to the question of what students should be advised to take down as notes on a blank piece of paper.

Zhou et al. (2022) assigned three groups with three different ways to take notes: in Chinese, in English, and in a combination of all languages, symbols, drawings, etc., respectively [7]. The results are that a free style of languages, symbols, and drawings outperformed either the use of L1 or L2, showing much room for a flexible use of content.

Siegel (2020) designed a four-step experiment for L2 learners to practice note-taking skills: (1) chunking with the transcript with a slash, (2) marking the transcript (using symbols, such as circles, triangles, stars, etc., before comparing with others, (3) writing verbatim notes, and (4) simplifying notes [21]. By also studying a control group with no explicit instruction, he proved that explicit instructions like this will have a significant impact on students’ performances. In this success design, it cannot be ignored that signs occupy half (step 1 and 2) of the content of these four steps and the format is not taken in as a crucial element.

In a recent case, however, Siegel acknowledges that “symbols such as #, =, @ can be useful” [9] (p. 248) but he does not consider that they contain much semantic information for content words as well as an indication of a relationship. In his previous trial, verbatim is step (3) [21]. The question arises as to what can be done if L2 learners are unable to understand the words they hear, thus making verbatim impossible. Further questions are then begged: whether signs can be of help and whether marking the relationship between words can improve understanding of a lecture or a listening task.

While Bui and McDaniel (2015) innovatively compared formats/structured notes with diagrams [19], some researchers have begun to adopt a more integrated way that combines formats and special contents together as the guide for note-taking.

For EFL learners, one other approach to note-taking is Educational Testing Service (ETS)’s TOEFL Good Notes Guidance (GNG). GNG includes seven segments: General, Language, Content, Efficiency, Accuracy, Organization, and Review of Notes. In a study of GNG, P. L. Carrell [9] (p. v) indicates that a brief intervention implementing GNG at the beginning of a TOEFL test had only limited effectiveness and little correlation with improved listening performances. At the same time, according to Carrell [9] (p. 5), researchers have found a positive correlation between the usage of abbreviations and icons with improved comprehension scores. The question that remained unsolved with this study is as follows: will a longer duration of experiment with this integrated approach work?

In sum, except for the GNG example, in most of the above-mentioned cases, students were *given* something to work on, either an organized format, a relevant diagram, or even a transcript. Once again, here comes the question: in real exam situations, such as TOEFL or the nation-wide “CET-College English Test” in China that has millions of examinees (10 million in the year 2017 alone), no aid will be given on the spot. How should teachers prepare students to help themselves with note-taking?

### 1.2. The Design of SBN Based on Signs and the Principles of Iconicity

Since symbols, abbreviations, and icons, etc., have been proved to help promote better comprehension [7,14,21,22]—and early language learners usually viewed letters (abbreviations) as image icons [23] (p. 346)—it is reasonable to expect that icons can be effectively substituted for words, phrases, and sentences in note-taking. It is further expected that icons could be good enough for effective note-taking, since it can be modified to symbolize words and relations. Carrell’s findings on GNG, together with the aforementioned positive correlation, thus became the primary motivation of this research, wherein icon (sign)-oriented guidelines for note-taking (SBN) were created in order to gauge the effectiveness in improving listening comprehension.

Modern semiotics recognizes three kinds of signs: icons (forms representing real objects), symbols (ubiquitous and conventional form/meaning relationship), and indices (the positioning of signs in terms of space and time) [24] (p. 300). Despite their distinguishing characteristics, the uses of all three types often overlap. Through the use of icons, as well as symbols and indices, similarities between linguistic forms and meanings can be easily understood. For an icon example, the Chinese character 人 (person) is an actual picture of the profile of a walking person. For an example of a symbol, greater than (‘>’) and the smaller than (‘<’) mathematical signs indicate comparative quantitative values by positioning the open or the closed side, respectively, of the symbols next to the value being compared. Finally, an example for indices would be that of a weather vane. The visible direction of the vane is the index of the direction of the wind which is invisible. Similarity, association, and conventionality are key concepts in the usage of the three kinds of signs.

The organization of signs involves three principles of iconicity: iconic sequencing, proximity, and quantity. The main idea of iconicity is about how languages reflect people’s perception of the world. Iconic sequencing is the placement of signs in accordance with the order in which events/objects occur. Iconic proximity is represented by the amount of distance between signs that separate one event/object from another. Iconic quantity is the size or the number of given signs representing amount or degree of information [24] (pp. 301–305).

SBN, therefore, was created by implementing the three types of signs as the content of notes (see Table 1) and organizing the notes based on the three principles of iconicity (see Table 2).

### 1.3. Research Objectives and Questions

To conclude from the previous studies, especially those in 1.1 and 1.2, several research gaps are open for discussion. First, in terms of research design: there is a dearth of relevant empirical comparative studies with a control and an experimental group, and also a lack of replication studies [11,21]. Moreover, while there has been comparison between notetaking formats vs. no-format, explicit instruction vs. no explicit instruction, symbols vs. no-symbols, etc., there remains a need for a comparison between different notetaking methods that use symbols. Second, in terms of the use of symbols: there seems enough room for symbols to represent not only relationships but also content words, but the question arises as to how well and why can SBN be applicable. Third, in terms of addressing L2 learners’ deficiency: what should they do when there is unknown information or unknown words? How well can SBN help in this case?

To address the above-mentioned previous research gaps, this study implemented a learner-centered 3 × 3 design for note-taking that is compatible with current teaching methodology, and is intended to test the following questions:

1. Would the SBN intervention result in better performance that is statistically significant compared to GNG and the traditional approach?
2. Would a longer intervention with GNG generate statistically significant improvement on performance?
3. Would the results of the three interventions be impacted by the change of an instructor?

## 2. Materials and Methods

### 2.1. Participants

Three (out of six) complete first-year classes of 32, 33, and 34 students were selected after a pre-test. A one-way ANOVA test showed no significant difference among them in listening performance prior to intervention: F (2, 98) = 0.449, *p* > 0.05. Participants were aged 18–21. Almost half of the participants were female, so gender difference was not considered a variable in this study.

The two instructors of these classes designed and conducted this research. Both hold MAs in Linguistics and have taught at the university level for more than ten years. One instructor taught and guided all three classes in the first six weeks of intervention, while the other instructor observed the classes. In the latter ten weeks of intervention, they reversed roles. One experienced EFL professor supervised the whole process.

### 2.2. Materials

#### 2.2.1. Listening Materials and Comprehension Tests

In a Chinese EFL listening environment, the closest thing to a mini-lecture is a Listening Passage (LP). Usually consisting of several passages, and lasting about three minutes, an LP requires students to answer two to five multiple-choice questions. In textbooks and most authoritative exams, LPs are ubiquitous and potentially problematic. LPs are read only once; the questions are also read only once, immediately after the LPs. Neither questions nor a transcript of the LPs are printed on the exam. Questions involve both the general ideas and the specifics of LPs.

In each class, LPs were used as listening material. Pre-tests, post-tests, and delayed post-tests all had three LPs and nine multiple-choice questions. Post-tests were again used as delayed tests. Each question was valued at one point and the final scores were converted to a 100-point scale. A total of 38 LPs were randomly selected from a bank of 48 which were drawn from the official biannual College English Test (CET) of the past 10 years. Original CET recordings were used, and accents and speech rates were both standard and applicable to lower-intermediate EFL students. Topics covered a broad range of subjects, from humorous stories to scientific reports.

#### 2.2.2. A Pre-Intervention Questionnaire

Before the intervention, note-taking needs analysis questionnaires were given to 60 students, 20 from each group. Students were asked which one of the five listening strategies (1. Prediction; 2. ‘Listen out’ for key words; 3. Monitoring and Evaluating; 4. Using clues; and 5. Note-taking) they would consider using when both the LPs and questions were read only once during an exam. The first four have been advocated as playing an important part in the listening process [2] (p. 750). Among these given strategies, 97 out of 99 students chose to take notes.

#### 2.2.3. The Interview

A semi-structured interview protocol was designed to obtain qualitative data and to probe into participants’ experiences, feelings, and ideas about the use of different note-taking activities. After the delayed post-test, 22 students (11 from each EG group) were interviewed informally and individually with only one question:

*‘What is your opinion about GNG/SBN?’*

More reticent interviewees were also asked a follow-up question:

*‘What are the advantages/disadvantages of GNG/SBN?’*

By virtue of individual differences, each interview lasted approximately from 5 to 10 min. All interviews were voice-recorded.

### 2.3. Procedures

This research was conducted with mandatory classes for non-English majors at a Chinese university. In the three 16-week listening classes, students had a 100-min listening lesson per week. They were encouraged to take no extra English lessons so that the extent of their listening and general language learning opportunities were kept highly similar, as suggested by Cross and Vandergrift [25] (p. 86).

A pre-intervention needs-analysis questionnaire, a pre-test, and the intervention period were conducted in sequence. After six weeks, a post-test was administered to test short-term effects. At the end of the 16th week, a delayed post-test was administered preceding the interview. Pre-, post-, and delayed tests have only objective multiple-choice questions. The instructors scored the tests and double-checked the answers to the questions, as well as the responses to questionnaires and interviews.

All groups were instructed by the same two instructors using similar material, syllabi, and timing for each step of the procedure. The teachers also practiced the same listening strategies with all three classes: Prediction; ‘Listen out for (key information)’; Monitoring and Evaluating comprehension; and Using clues. Despite these commonalities, the three groups were differentiated by the different note-taking strategies that were taught, emphasized, and practiced. CG students were only instructed to ‘take notes,’ and more often followed the traditional approach of repeated listening. EG1 was allowed more time to understand and practice only GNG. EG2 engaged in explanation and practice of only SBN.

### 2.4. Data Analysis

To address the research questions, both quantitative and qualitative data were collected and analyzed. The pre-, post-, and delayed post- tests results yield statistical data in relation to research questions 1 and 2; the pre-intervention questionnaires yield quantitative results that help to consolidate the rationale of the design and experiment of 3 different kinds of note-taking; the one-on-one interviews produce qualitative data.

Quantitative data: Test results were analyzed using one-way ANOVA, paired-sample tests, and a 3 (group) × 3 (time) design Repeated Measure ANOVA in the SPSS Statistics 26.0 software package. First, one-way ANOVA tested to what extent each group performed differently in each test (pre-, post-, delayed post- tests). The one-way ANOVA results for pretest also tell whether the students of the three groups are distinctly different regarding their English levels, which is *Not*. Second, paired sample tests were implemented to see if each group (CG, EG1, EG2) has improved with time. Finally, The Repeated Measure ANOVA was adopted to test if there is an overall and significant difference with the 3 × 3 design. These three tests allowed researchers to examine whether there were statistically significant differences among the three groups’ performances over time.

Qualitative data: This study conducted semi-structured interviews to triangulate the quantitative results and to obtain a fuller and deeper understanding of the perceptions and feelings of the participants. The recorded interviews were transcribed, read through, and double checked by both interviewers. Next, the verbatim transcriptions were then coded using *advantages*, *disadvantages*, and *future perspectives*. A constant comparative method was used to note similarities and differences within each of the 3 categories across all interviewees. The findings are noted below.

## 3. Results

### 3.1. Quantitative Findings

**Figure 1:**
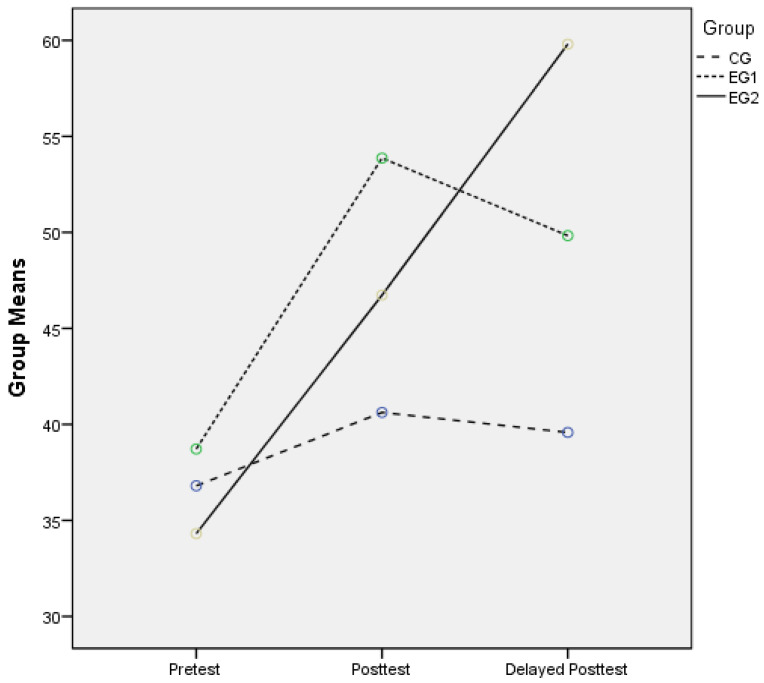
Group means on multiple-choice tests scores over time.

The descriptive statistics for the multiple-choice tests, including group means and standard deviations for each group over time, appear in Table 3. The group means are plotted on the graph in Figure 1.

Concerning GNG, results demonstrate that significant improvement was achieved through a longer and more detailed intervention than that tested by Carrell. At the delayed post-test juncture, GNG effectiveness decreased with an instructor change, indicating that GNG’s effects are largely dependent on the instructor variable.

With respect to SBN effects, Table 3 and Figure 1 illustrate that EG2 improved significantly over CG and EG1, suggesting that SBN outperformed GNG and the traditional approach on the delayed post-test after treatment over time (F = 7.578, *p* = 0.001). Similarly, when the second instructor took over, CG and EG1 showed slight, albeit evident, decline at the delayed post-test juncture; nevertheless, despite this instructor change, EG2 maintained improvement. This confirms that SBN produced superior performance and was less susceptible to instructor influence.

### 3.2. Qualitative Findings

Post-interview results from students’ comments of the perception of the EG interventions can be grouped into three categories of advantages, disadvantages, and future perspectives.

#### 3.2.1. Advantages

Both sets of EG interventions (GNG and SBN) received positive feedback. Students considered them ‘good’ because they expressed that they ‘know how to take notes now’, whereas in the past they did not.

Most students in EG1 said GNG is ‘clear’, ’helpful’, and ‘detailed’. EG2 students, however, provided more feedback than EG1. They commented that SBN was ‘well-organized’, ‘fun’, ‘reasonable’, ‘enlightening’, and gave them a feeling of receiving ‘new knowledge beyond English’. Additionally, students stated that ‘the whole logic and flow of the passages are clearer’ and ‘it’s easier to spot answers or guess correctly’. Additionally, some students who were once afraid of L2 listening claimed that they feel more confident about LP now. Comments on SBN outnumbered those for GNG and were more diverse.

#### 3.2.2. Disadvantages

Both EGs shared the perception that the note-taking instructions they were given were difficult to practice. They did not believe they ‘can totally learn the strategy well’.

For GNG, the problem was with lengthy, difficult-to-grasp explanations. A six-page GNG handout clearly exhausted the students. ‘Too many’ and ‘a little bit messy’ were the most common comments from EG1. For SBN, the problem was that many students felt that there was still ‘not enough instruction’. SBN students claimed that ‘it seems impossible to remember all signs for all words’.

#### 3.2.3. Future Perspectives

Feedback from the two EGs was different in a rather fundamental sense. In the case of GNG, what students wanted was more time and practice. In the case of SBN, the desire was for something concrete, such as: an SBN handbook; ‘a more systematic’ or ‘personalized’ set of guidelines; a ‘booklet of signs’; or a ‘most common CET vocabulary list of signs’.

## 4. Discussion

This study investigated whether SBN is more effective than GNG and the traditional methods in improving comprehension performance in classrooms, and whether GNG improves over time. The results are positive and are in accordance with the aforementioned positive correlation of icons with improved comprehension [15] (p. 5). SBN brought about significantly superior performance over time, regardless of the instructors’ influence, and GNG improved performance over about a month’s time.

To conclude, the significance of the differences of the three methods is threefold. First, the SBN and GNG participants had yielded significantly higher scores in the posttests than the traditionally trained participants. In the results of the delayed post-test, their performances changed, while continuing to be significantly different, with the SBN group continuing to improve while the other groups both descended. Second, the statistically significant differences displayed from the post-test to delayed post-test reveal that the SBN method is efficient enough to not be subjective to the instructor’s influence, unlike the other two groups. Third, compared to the GNG group, the SBN participants produced more feedback, and they communicated interests in SBN and a desire for more training, which illustrated that SBN has aroused their interest in SBN and strengthened their engagement in note-taking.

Aside from a needs analysis, which facilitated learners’ autonomous awareness and oriented this study towards a ‘diagnostic’ approach, as suggested by [26], four cognitive principles underpin the results.

### 4.1. The Effects of Meaningful Elaboration

Cognitive psychology confirms that memory favors ‘Meaningful Elaboration’ most, followed by ‘Elaborative Processing’. Some research has been designed in the L2 grammar and lexical acquisition and proving “elaborative processing” effectively promoting students’ L2 abilities [27,28,29]. Meaningful elaboration has received even fewer notifications in L2 acquisition.

However, not so much in the field of L2 Listening acquisition has been attempted. In a listening process without the interference of visual material, an attentive mind forms audio memories. If students take notes, they then acquire visual memories along with audio memories. Reportedly, audio-sensory memory lasts only up to 10 s [30] (p. 150), which is sufficient for processing and taking brief notes, but not for accurate subsequent retrieval of information. Visual memories also hold information briefly. Unless information is attended to and processed further, it will be lost [30] (p. 149). How, then, can students ‘attend to’ and ‘process’ the information to extend memory retention and improve information retrieval? More Elaborative Processing results in better memory [30] (p. 166). Elaborative Processing means offering additional information relating to and expanding on what was presented and needed to be remembered. In second language acquisition, a typical example is the aforementioned advance organizer, which includes guided notes. These organizers have been repeatedly researched in both reading and listening studies, and they are a type of Elaborative Processing with text materials. It is to be noted that these advance organizers tend to offer students guides in which the elaboration comes more from an external source.

Meaningful Elaboration, on the other hand, comes from within the students. Both SBN and GNG tend to foster in students the ability to create Meaningful Elaboration, which Anderson distinguishes from elaborative processing [30] (p. 169). Whereas Elaborative Processing activates performance, Meaningful Elaboration generates creative additions to the information provided. Activities, such as brainstorming for a synonym of a given word, or, as in our study, creating a sign based on information heard, benefit memory, and create Meaningful Elaboration.

Meaningful Elaboration, therefore, accounts for the significant improvement produced by SBN and GNG, in which, instead of being offered help, students were required to actively generate more efficient elaboration for memories, thus evoking higher levels of activation than shallowness of elaborative processing [30] (p. 170). Moreover, SBN’s creative process of signs, with the more concrete aim of being ‘Similar’, ‘Associative’, ‘Conventional’ and ‘Orderly’, adds additional clear-cut processing requirements to Meaningful Elaboration than GNG. This, too, may contribute to the better performance of SBN over GNG. This increasing linear amount of improvement echoes the greater diversity in feedback from the SBN interviews.

### 4.2. The Effects of Cognitive Prominence

The second principle is a sequence of cognitive priority on the basis of Prominence View in cognitive linguistics. Fischer defined Prominence View as that which is concrete takes priority [23] (p. 345). Therefore, what is concrete, obvious, active, and special catches attention better than the abstract, obscure, static, and ordinary. Formatted signs feature likeness and association, and emphasize order and flow of information and events. They are more concrete and active, thus being more prominent than abstract and static content words. As concluded, children are linguistic icon-makers and they interpret many things in iconic ways [23] (p. 345). Lower-intermediate L2 students, who in many ways resemble children starting to acquire a language, also seem to process iconicity-focused SBN better. Their cognitive preference for iconic information further accounts for the surpassing performance of SBN over GNG. Psychologists have found similar effects in L2 speech acquisition: with enough help, L2 learners can show native-like processing of prominence [31].

The focal effect of cognitive prominence has been repeatedly proven by eye-tracking experiments in the field of visual working memory. Neuroscientists have found that participants’ ability to recall an object’s spatial location is positively correlated with the object’s salience/prominence, which strengthens with increasing task difficulty [32]. Accordingly, if both distractors and the aimed objects in a memory task are controlled to display a certain extent of salience, it is possible to show that when the distractors are made to appear more salient, the participants’ success of task completion will be undermined [33]. In our study, therefore, while the control group are making efforts to collect meanings from mostly their verbatim notes, the two experimental groups benefit from the use of notes that capture those salient objects which have been made prominent during the process of listening. Whilst the group using GNG must contend with notes built on complicated guidelines—with a certain amount of signs, as directed—the SBN groups are able to achieve improved recall from a greater number of signs based on the iconicity principles, hence achieving better results in the listening comprehension task.

The manipulation of both the auditory and the visual systems relies on an effective executive functioning. Perhaps the working memory model that can most directly relate to and best explain the SBN group’s effective executive function regarding information processing is still the classic Multi-Component Model of Baddley and Logie, which is composed of both a supervisory system (the central executive, which is not equipped with the supplementary storage capacity) and the specialized temporary memory systems, including a base of a “phonological loop”, and a “visuospatial sketchpad” [34] (pp. 28–30). For subjects in the SBN group, the central system receives information from the phonological loop (on the listening task), and individuals may shift their attention from the auditory system to the visual–spatial sketchpad, thereby translating attention into an individual and/or a sequence of salient sign(s) which have already been rendered conveniently convertible for SBN note-taking. This model is most exploited in L2 acquisition, though observed mostly through a traditional psychological lens [35]. Cowan’s model [36] that recognizes the impact of professional knowledge on the consolidation from working memory to long-term memory may account for the difference in performance between the groups using GNG and SBN, respectively, as both groups were trained with different (diverse and demanding vs. focused and flexible) note-taking skills.

### 4.3. The Effects of Integration and of the Gestalt Principles

The third principle is that a modified bottom-up activity interacts more effectively with top-down strategies. While GNG does not emphasize similarity between the layout of the signs and the order of events or objects, SBN works more effectively toward the creation of a more sophisticated gestalt (complete picture) for the aforementioned top-down strategies to work with. For Prediction, because SBN does not advocate a ‘key word’ approach, which is usually beyond the capacity of lower-intermediate students, signs help listeners to keep track of the information provided so that they can predict with fewer inaccurate memories and less anxiety. For ‘Listening out for (key points)’, instead of feeling desperate for ‘keys’, students were relaxed enough to be able to take down whatever they understood. For Monitoring and Evaluating, students were already monitoring their listening activities during the creation process of a well-structured logical system of signs. Checking signs helped them to evaluate the listening process with visible products of their own creation. Furthermore, with a question mark in the middle of a context of other signs, students were provided with a more easily accessed gestalt to speculate on, or to infer, the meaning of the words that they had missed, as a gestalt gives rise to more information than the sum of its parts [30] (p. 8).

Consequently, the notes that the students made became a well-structured picture of signs with the questioned information highlighted; an attempted ‘whole picture of events’; a representation of listening material in the forms of formatted signs; and a gestalt of Meaningful Elaboration. The gestalt produced a vivid portrait of the listening text; it provided a more detailed, active, and logical bottom-up platform for the top-down approaches to work with, analyze, and examine the information heard, thereby allowing students to achieve higher performance in comprehension. The gestalt phenomenon, in which we perceive more than is actually presented, has been repeatedly tested in speech perception when the brain automatically generates a missing pitch, in translation appreciation when linguistic organization and visualized scene naturally merge into a gestalt [37], and in oral comprehension when the visual and auditory information were integrated as a helping gestalt [38].

Besides the four principles, the test results also indicated that instructors’ influence had no impact on note-taking effectiveness in SBN. Perhaps it was because the guidelines were simple and did not require students to exert an effort to ‘listen out for’ the key words, thus alleviating pressure on them and preventing them from becoming too dependent on the instructor.

### 4.4. Pedagogical Implications

As this research demonstrates, because SBN was proved effective, and its guidelines are simpler and easier to implement, it seems possible to use this as an alternative to GNG, with more examples but fewer words to read. Many textbooks on interpretation incorporate a section of shorthand signs. Handbooks for SBN could do the same thing. While SBN is being researched for its effectiveness, implemented in more contexts, and support materials such as guides undergoing development, other sources such as interpreting shorthand guidance, iconicity readings, and even GNG may all work as a primitive SBN alternative.

Furthermore, since only 2494 words appear ‘most frequently’ in current English text, regardless of topic, genre, and location [39] (p. 1), it is conceivable that common signs can be created for these words and given to students for reference. Many words can be grouped as synonyms and antonyms, sharing similar signs.

Furthermore, while students are happy using the Chinese iconic characters in this study, international students can be encouraged to employ icons in their own culture as notes as well, since the translanguaging method is on the rise for note-taking [5,7].

### 4.5. Limitations and Future Research

By having students ‘draw out the whole picture’, the research showed that SBN strategies compensated somewhat for students’ lack of vocabulary knowledge and frequency of breakdowns in understanding during listening. To address these issues, students were asked to jot down a question mark and to infer from the gestalt; however, not much else has been done to solve these basic problems. This was reflected in the interview responses, such as ‘I just don’t know the word’ and ‘sometimes I just don’t understand’. Some research pertaining to these issues has been attempted [40,41]. Future studies should include these cognitive approaches. Further, the notes that students took may also be a subject of close examination, which might shed light on exactly what the listeners do not understand and how to help them confront this problem with better notes.

We must also acknowledge that LPs are only one kind of listening material. More authentic materials, as well as new types of material, for example news videotexts, which were considered by Cross to be the ‘holy grail’ of L2 Listening comprehension [42] (p. 151), should be targeted and tested. More test types, such as lectures, conversations, and blank-fillings, should also be added to test the effectiveness of SBN. This future orientation might also answer the “not enough training” disadvantage that some students mentioned during the interview session.

## 5. Conclusions

Based on semiotic sign structure and iconicity, designed for a simple but detailed note-taking guide for L2 Listening, SBN emphasizes the cognitive process in listening and note-taking and encourages students to draw ‘a whole picture’ of signs for top-down strategies to work with. In the SBN creation process, Meaningful Elaboration and prominent icons helped to provide a vivid gestalt that memory and cognition favor. Whereas more cognitive research needs to be conducted to overcome the shortcomings, SBN has been proven effective in L2 Listening classrooms for instructors to teach and for students to learn. Signs somehow make up for the deficiency when intermediate level L2 learners fail to recognize some words during listening. The instructor variable did not affect SBN results, and students expressed their desire for more SBN instruction as well as their confidence for improvement in L2 Listening.

## Figures and Tables

**Table 1:** Content of SBN notes—three types of signs.

	Types of Signs	Relationship with the World	Examples and Explanations		
			Passage Heard	Note Examples	Explanation
1	icon	Similarity	‘Andorra, one of the smallest countries in the world, is located high in the mountains between France and Spain.’	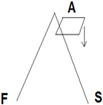	A and the parallelogram represent ‘Andorra’; arrow downward refers to its being the smallest country; and the two lines represent mountains.
2	symbol	Conventionality	‘He loves to listen to music.’	♂♡♪	These symbols are borrowed from other fields to represent a male subject, love, and music.
3	index	Association	‘Mom was instantly furious’	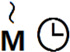	M represents ‘Mom’, the vertical tilde pictures fuming, and the two-stroke clock sign depicts time.
plus	question mark			?	Question marks represent when unknown information occurs.

**Table 2:** Organization of SBN notes—three iconic principles.

	Organization/Iconicity Principles	Relationship with the World	Examples and Explanations		
			Passage Heard	Note Examples	Explanation
1	Iconic Sequence	order/logic of signs = order/logic of objects/events	‘Event A happened because of Event B.’	Event B → Event A or ∵Event B ∴Event A	causal relationship
			‘This event lasted for 10 years.’	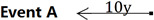	time sequence
2	Iconic Proximity	number of signs = amount/number of objects/events/concepts	‘While the female…, the male…’	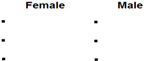	information clusters representing idea/object clusters
3	Iconic Quantity	distance between signs = distance of objects/events	‘The manager makes more money now.’	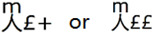	‘More money’ is represented by the plus sign of ‘+’, or double signs of money. (The letter ‘m’ above ‘人’ refers to the manager, and ‘人’ is a Chinese character of ‘person’ or ‘people’.)

**Table 3:** Group means and standard deviations for multiple-choice tests.

	Pre-Test		Post-Test		Delayed Post-Test	
Groups	M	SD	M	SD	M	SD
CG (n = 32)	36.8	17.95	40.62	17.54	39.58	19.12
EG1 (n = 33)	38.71	20.81	53.87	19.47	49.83	21.18
EG2 (n = 34)	34.31	18.43	46.73	14.42	59.8	14.22

## Data Availability

The data supporting the findings of this manuscript are available from the corresponding author upon reasonable request.
